# A review of terahertz phase modulation from free space to guided wave integrated devices

**DOI:** 10.1515/nanoph-2021-0623

**Published:** 2021-12-19

**Authors:** Hongxin Zeng, Sen Gong, Lan Wang, Tianchi Zhou, Yaxin Zhang, Feng Lan, Xuan Cong, Luyang Wang, Tianyang Song, YunCheng Zhao, Ziqiang Yang, Daniel M. Mittleman

**Affiliations:** Sichuan Terahertz Communication Technology Engineering Research Center, School of Electronic Science and Engineering, University of Electronic Science and Technology of China, Chengdu, China; Yangtze Delta Region Institute (Huzhou), University of Electronic Science and Technology of China, Chengdu, China; Shijiazhuang Communication Measurement and Control Technology Research Institute, Shijiazhuang, China; School of Engineering, Brown University, Providence, RI, USA

**Keywords:** metamaterials, metasurface, phase modulation, terahertz modulators

## Abstract

In the past ten years, terahertz technology has developed rapidly in wireless communications, spectroscopy, and imaging. Various functional devices have been developed, such as filters, absorbers, polarizers, mixers, and modulators. Among these, the terahertz phase modulation is a current research hotspot. It is the core technology to realize flexible control of the terahertz wavefront, beam scanning, focusing deflection. It is indispensable in terahertz wireless communication, high-resolution imaging, and radar systems. This review summarizes the research progress of terahertz phase modulators from the two major types: free space and guided wave integration. Among these, the free space terahertz phase modulator is realized by combining the tunable materials and artificial metasurfaces. Based on different types of tunable materials, the terahertz free space phase modulator combining the semiconductor, liquid crystal, phase change materials, graphene, and other two-dimensional materials are introduced, and the influence of different materials on the phase modulation performance is discussed and analyzed. The monolithic integration and waveguide embedding methods are introduced separately, and the characteristics of different forms of terahertz-guided wave phase modulation are also discussed. Finally, the development trends of terahertz phase modulators, possible new methods, and future application requirements are discussed.

## 1 Introduction

Terahertz technology has shown significant development potential in data communications, radar detection, high-resolution imaging, and other fields. However, these applications also face the problem of short transmission distance caused by atmospheric absorption and free-space path loss [1], [2], [3]. The primary method for managing this problem is increasing the antenna gain to compensate for these losses. However, most high-gain antennas are not easily steered, so the propagation angle is fixed, which reduces the practicability of the system. As one solution, terahertz wavefront shaping technology can flexibly manipulate the beam direction and wavefront characteristics to meet the application requirements of terahertz systems. For example, the terahertz beam directivity can be improved in a point-to-point communication system to increase the propagation distance. Multi-beam wavefront characteristics can be switched to achieve multi-area detection in target radar systems [4, 5]. The wavefront mode mask can be switched to provide the system with more perceptual information in terahertz high-resolution imaging [6], [7], [8]. To achieve these goals, terahertz wavefront shaping requires synthesis by multiple channels carrying suitably variable phase signals. Therefore, terahertz phase modulation devices have become one of the most critical technologies for terahertz systems.

The phase of the electromagnetic wave can be characterized by the equivalent phase constant and the transmission distance [9]. After an electromagnetic wave with an initial phase of $\varphi_{0}$ propagates in material or medium with an equivalent phase constant of *β*, its phase φ generally is expressed as $\beta l+\varphi_{0}$ . Therefore, changing the equivalent phase constant or propagation length can cause the phase to change. The strategy of changing transmission distance is widely used in microwave frequency bands [10, 11]. For example, switching line phase shifter, which is based on the idea that diodes are used as switches to select transmission lines of different lengths to control phase shift. However, this strategy needs to add additional structures on the basis of the original structure, which increases the complexity of the modulator and increases the parasitics. Another strategy more suitable for the terahertz band is changing the equivalent phase constant, which is related to the electromagnetic properties of the material or the dielectric properties of the medium. A free-space terahertz phase modulator (FSPM) basically applies changes in the resonance characteristics of metamaterials to change the phase constant to modulate the phase.

As early as 2000, Kersting, Strasser, and Unterrainer proposed the first THz phase modulator [12]. This early report described a semiconductor device which permitted electronic control for modulating the terahertz phase, opening the door to the research on terahertz phase modulation. Subsequently, a free-space terahertz phase modulator combining GaAs and split resonant ring (SRR) was proposed by HT Chen [13]. The applied voltage controls the carrier concentration of GaAs at the gap of the SRR, thus controlling the transition of the resonance mode to modulate the terahertz phase. The team also focused on explaining the changes in the phase of the terahertz wave in connection with the amplitude changes. Experiments proved that the amplitude and phase satisfy the Kramers–Kronig (K–K) relationship, which provides theoretical guidance for later research on terahertz phase modulation [14]. Subsequently, numerous terahertz phase modulators with different methods, materials, and structures have been proposed and demonstrated. The basic principle is to change the electromagnetic characteristics of the semiconductor materials, phase change materials, liquid crystal materials, graphene, or other materials. These materials can be combined with a custom-designed metasurface to change the resonance characteristics of the meta-atoms, thereby realizing the modulation of terahertz waves [15], [16], [17], [18], [19]. This kind of FSPM can be divided into transmission type and reflection type, most often in a quasi-optical configuration. Transmissive phase modulation means that electromagnetic waves directly pass through the metasurface to modulate the phase. Reflective phase modulation means that electromagnetic waves illuminate the metasurface and reflect back in free space with modulated phase. In contrast to free-space devices like the FSPM, guided wave phase modulators (GWPM) modulate the phase of a terahertz-guided wave in a waveguide or on chip. This modulation principle is completely different from the principle of the FSPM and can be divided into two categories: a monolithic integrated phase modulator (MIPM) and a waveguide-embedded phase modulator (WEPM). The former mainly uses two orthogonal signal vector syntheses, while the latter mainly changes the equivalent propagation constant in the waveguide.

**Figure 1:**
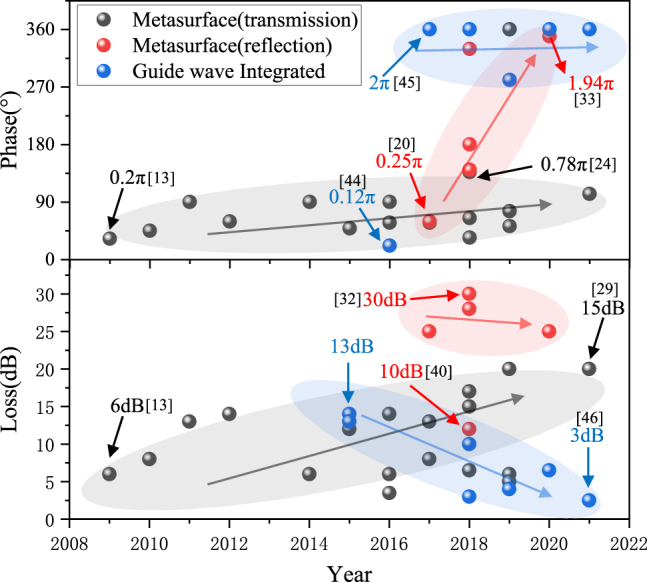
Recent advances in terahertz phase modulators.

Typically, phase modulation range and insertion loss are key performance metrics for phase modulators. Over the past decade, many outstanding results have been reported. As shown in Figure 1, the terahertz phase modulators have evolved rapidly, with the modulation phase increasing from 36° to 360° and device loss decreasing to below 3 dB. Different modulator types have different trends, such as transmission-type FSPM whose modulation phase increases from 36° to 137°, and the loss increases with the increase of modulation phase, and reflective-type FSPM whose modulation phase can reach 360°. However, its insertion loss is large (≥20 dB). Meanwhile, the GWPM’s loss decreases gradually while ensuring a significant modulation phase. Some representative results of the terahertz phase modulator are shown in Table 1. Modulators based on different materials have different characteristics. For example, phase modulators based on doped semiconductors are easier to fabricate and can be switched by external light or electric field. Phase modulators based on two-dimensional electron gas or two-dimensional materials can exhibit fast modulation speed and are amenable to integration. Liquid crystal modulators also have great potential for phase modulation, balancing the (sometimes large) insertion loss. These different types of phase modulators provide a wealth of ideas for terahertz phase modulator designs. For example, the transmission FSPM is simple in design but usually has a smaller phase modulation, while the reflection FSPM has a larger phase modulation but the insertion loss is generally large. GWPM has larger phase modulation and moderate insertion loss. Although GWPM has excellent performance, it still cannot replace FSLM. The difficulty of the process increases the cost, and some novel functions cannot be implemented by GWPM, such as spatial light modulation, compression sensor modulators and dynamic wave plates for imaging. Therefore, in this review, we consider both terahertz FSPM and GWPM, discussing representative examples of each. The second section reviews the development of terahertz FSPM in recent years. According to the type of tunable material, modulator combined with doped semiconductors, liquid crystal materials, phase change materials, graphene, and other two-dimensional materials are introduced separately. Section 3 reviews the development of terahertz GWPM in recent years and introduces two types of monolithic integration and waveguide embedding. Section 4 reviews part of the functional devices derived from the terahertz phase modulation. Section 5 summarizes the advantages and disadvantages of terahertz phase modulators and looks forward to the future practical application scenarios and development trends.

**Table 1:** Terahertz phase modulator types and basic performance.

Time	Types	Frequency	Phase	Loss	Rate	Reference
2009	Metasurface with doped GaAs	0.88–0.9 THz	32.4°	6 dB	30 kHz	[13]
2010	Metasurface with doped GaAs	1–1.25 THz	45°	8 dB	–	[20]
2011	Metasurface with doped Si	0.5–1.7 THz	90°	13 dB	–	[21]
2019	Metasurface with doped Si	0.8–1.56 THz	52.2°	6 dB	500 MHz	[22]
2015	Metasurface with HEMT	0.2–0.4 THz	48.6°	12 dB	1000 MHz	[16]
2018	Metasurface with HEMT	0.32–0.37 THz	136.8°	15 dB	–	[23]
2017	Metasurface with HEMT	0.7-1 THz	57.6°	13 dB	2.7 MHz	[24]
2016	Metasurface with vanadium dioxide	0.77 THz	90°	14 dB	–	[25]
2016	Metasurface with vanadium dioxide	0.1 THz	57.6°	6 dB	–	[26]
2018	Metasurface with vanadium dioxide	0.37–0.52 THz	64.8°	6.5 dB	–	[27]
2021	Metasurface with vanadium dioxide	0.63–0.79 THz	102.6°	20 dB	–	[28]
2018	Metasurface with vanadium dioxide	0.57–0.63 THz	136.8°	17 dB	–	[29]
2018	Metasurface with liquid crystal	0.34–0.36 THz	360°	28 dB	1 kHz	[30]
2018	Metasurface with liquid crystal	0.32–0.34 THz	329.4°	30 dB	142 ms	[31]
2020	Metasurface with liquid crystal	0.08–0.12 THz	349.2°	25 dB	–	[32]
2018	Metasurface with liquid crystal	2 THz	34.2°	–	10 ms	[33]
2017	Metasurface with liquid crystal	0.7 THz	59.4°	25 dB	–	[34]
2019	Metasurface with liquid crystal	0.4–1.2 THz	75.6°	5 dB	1 kHz	[35]
2019	Metasurface with liquid crystal	0.2–1.2 THz	360°	20 dB	1 kHz	[36]
2012	Metasurface with graphene	0.64 THz	59.4°	14 dB	–	[37]
2018	Metasurface with graphene	1.2 THz	180°	50 dB	–	[38]
2018	Metasurface with graphene	0.5–1.6 THz	140.4°	10 dB	10 kHz	[39]
2014	Metasurface with liquid crystal	0.2-1 THz	90°	6 dB	1 kHz	[40]
2015	Monolithic integrated with InP	0.22–0.32	360°	14 dB	–	[41]
2015	Monolithic integrated with InP	0.24–0.34 THz	360°	13 dB	–	[42]
2016	Waveguide integrated with MEMS	0.5–0.55 THz	21.6°	3.5 dB	–	[43]
2017	Waveguide integrated with vanadium dioxide	0.22–0.24 THz	360°	8 dB	–	[44]
2018	Waveguide integrated with artificial magnetic conductor	0.23–0.25 THz	360°	3 dB	–	[45]
2018	Monolithic integrated with SiGe	0.16–0.2 THz	360°	10 dB	–	[46]
2020	Monolithic integrated with SiGe	0.16–0.19 THz	360°	6.5 dB	–	[47]
2021	Waveguide integrated with MEMS	0.22–0.33 THz	360°	2.5 dB	–	[48]

## 2 Terahertz free-space phase modulator

The first metamaterial-based terahertz phase modulator was proposed by Chen et al. [10]. Their device utilized the electromagnetic reconstruction characteristics of metamaterials to enable phase modulation in the 10 s of kHz range. This work was extended by Mittleman et al. to achieve spatial light modulation, with a 4 × 4 pixel array, opening the door to modulate terahertz wavefronts in free space [49]. These early ideas were enabled by metamaterials, artificial structures with special electromagnetic properties. Early metamaterials mainly used continuously graded equivalent media parameters (equivalent dielectric constant and equivalent permeability) to simulate the electromagnetic properties of metamaterials. This analysis and design are mainly concentrated in the analogue domain, and the design process is more complicated and cannot be well integrated with digital information. With the development of metamaterials toward miniaturization and simplification, metasurfaces composed of a series of two-dimensional artificial structures have become a hot research topic. It retains the characteristics of realizing traditional metamaterials to control electromagnetic (EM) waves and compensates for the defects of large volume, high profile, and difficult manufacturing. The emergence of metasurfaces has brought some devices for electromagnetic wave manipulation, such as abnormal reflections, holography, polarization conversion, and beam focusing [50], [51], [52], [53], [54], [55], [56]. Research and analysis methods have been updated and iterated accordingly. In 2014, T. Cui of Southeast University proposed the concept of “digital metamaterials” [57], and the development of metamaterials entered the digital era. It digitally characterizes the phase response characteristics of metamaterials to electromagnetic waves in space. Specifically, the phase is modulated by applying an external bias voltage to the metamaterial unit. The number “0” represents the reflection/transmission phase 0, and the number “1” represents the reflection/transmission phase 180°. The combination of digital codes manipulates the electromagnetic wave similar to continuous metamaterials. The key to coding metamaterials lies in its “coding atom” (phase modulation unit), and its phase modulation ability determines the performance of the coding metamaterial. In the microwave frequency band, “coding atoms” are generally compounded by PIN diodes, varactor diodes, and artificial microstructures. However, since traditional microwave transistors no longer interact with terahertz waves, different types of semiconductor materials and phase change materials, liquid crystal materials, graphene, and other new materials combined with artificial structures are the main means for enabling terahertz FSPM.

### 2.1 FSPM compounded with doped semiconductors

**Figure 2:**
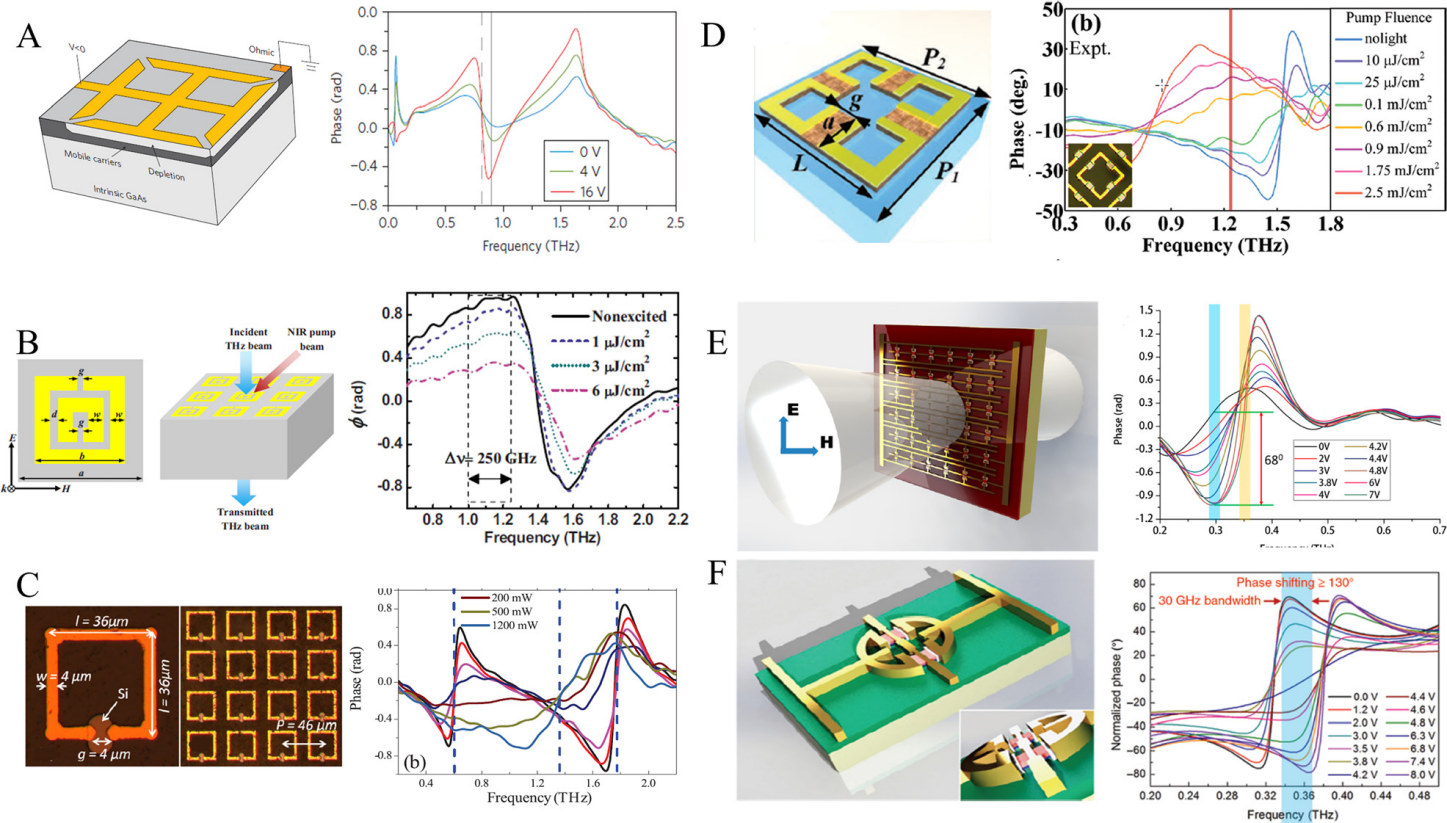
Structure diagram and phase modulation of doped semiconductor composite FSPM. (A) Terahertz phase modulator contains SRR and Schottky junction with an n-type GaAs layer and its phase modulation as a function of the applied voltage [13]; (B) schematic diagram of double SRR metamaterial fabricated on GaAs substrate and its phase modulation under different light intensity excitation [20]; (C) SRR structure composite with doped silicon and corresponding phase modulation [21]; (D) schematic illustration of the ultrafast tunable metasurface integrating photoactive Si bridges [22]; (E) double-channel heterostructure metasurface and its phase modulation [16]; (F) schematic diagram of the structure of the resonance-enhanced metasurface and its phase modulation results [23].

One basic principle of the FSPM is the switching of the resonance mode which causes phase modulation. The electric excitation changes the carrier concentration in the doped GaAs to cause the resonance characteristics to modulate the terahertz phase, proving to be a reliable method to regulate the terahertz wave, as shown in Figure 2A. In addition, light excitation to change the concentration of charge carriers has also been proved to be a reliable control method. In 2010, Manceau et al. proposed a light-excited metamaterial that consists of a split-ring resonator (SRR) array on a GaAs substrate, as shown in Figure 2B. By changing the intensity of the optical pump from 0 μJ/cm^2^ to 6 μJ/cm^2^, phase modulation of 45° is demonstrated with 250 GHz bandwidth [20]. Different from the previous work to change the conductivity of the entire substrate, in 2011, Roy Chowdhury et al. proposed to place the doped silicon as a switch in the middle of the SRR, as shown in Figure 2C. Dynamically controlling the photocarrier concentration in silicon by light illumination, the unit is converted from SRR to a closed-loop, which significantly changes the effective capacitance and thus achieves significant phase modulation up to 90° in the optical pump range of 0–1200 mW [21]. Recently, Hu et al. proposed a silicon bridge metamaterial with SRRs and doped Si composite nested to reduce conductivity and achieve ultra-fast phase/frequency tuning [22]. In this work, as shown in Figure 2D, the parallel silicon bridge is excited by light, and the four SRRs become a closed ring resonator. The basic magnetic dipole of the SRR is suppressed and converted into an electric dipole mode. Under 0.1–2.5 mJ/cm^2^ femtosecond pulse excitation, the demonstrated resonance frequency tuning range is expanded to 48% (0.81–1.56 THz) within 2 ns, and the phase modulation reaches 53.3°.

In contrast to the above cases where the carrier density of a bulk material is varied, a two-dimensional electron gas (2DEG) offers some advantages. This 2D carrier distribution can be induced by spontaneous piezoelectric polarization in a heterostructure. Utilizing a 2DEG, high electron mobility transistors (HEMT) have been developed and applied in many electronic devices, such as amplifiers, detectors, switches, and modulators. 2DEGs have extremely high electron mobility and carrier concentration, which is conducive to developing high-speed dynamic devices. In recent years, the application of 2DEGs coupled to metasurfaces in the field of terahertz high-speed modulation devices has shown extensive development. This scheme has the potential of an active dynamic composite metasurface for gate control of external circuits. Typically, nano-structured 2DEG components are embedded in a metal structure to construct an active metasurface HEMT array. Therefore, this structure is called a 2DEG metasurface. In 2011, the modulation of the terahertz wave amplitude by the 2DEG metasurface was demonstrated for the first time, reaching a modulation depth of 33% at 0.46 THz, with a switching speed as high as 10 MHz [58]. Following this work, Zhang et al. proposed a composite metamaterial terahertz modulator in 2015 based on GaN [16]. Compared with the second-generation semiconductor GaAs, GaN has a wide bandgap, electron mobility, and saturation speed. This work integrates an InAlN/AlN/GaN/AlN/GaN dual-channel heterostructure on an equivalent collective dipole array to break through the significant restriction to applying ultrafast terahertz active devices. Applying different voltage conversion dipole resonances, as shown in Figure 2E, achieves a phase shift of 68° and a modulation depth of 85%, with a modulation rate of 1 GHz. As a transmissive metasurface, this work has brought a huge leap in terahertz amplitude modulation, but the phase shift is still limited. Different from reflective metasurfaces, transmissive metasurfaces usually produce a larger phase shift by arranging multilayer structures in the microwave band. However, it is difficult to prepare multilayer metasurfaces in the terahertz region, let alone tunable devices. To solve this problem, Zhang et al. further proposed a single-layer HEMT metasurface in 2018, which dramatically increases the modulation phase of the terahertz wave by enhancing the resonance [23]. According to the K-K relationship, the proposed Inductance Capacitance Dipole Resonance (LCDR) produces a large resonance intensity to induce a large phase jump. By embedding GaN-HEMT in the LCDR resonant structure, the GaN-HEMT is used as a switch to dynamically control the surface current distribution and change the resonance state under different voltages. Experimental results show that this device has a phase shift of up to 137° (Figure 2F). In other HEMT metamaterial work reported in 2017, Zhou et al. focused on increasing the resonance intensity or improving the modulation depth and reducing the operating voltage of the existing devices. Based on the high resonance depth of the double SRR, the authors introduced HEMT into the quadruple SRR to enhance the resonance intensity. With only -4v gate voltage, the phase modulation is up to 38°, the modulation depth is about 80%, and the modulation speed is beyond 2.7 MHz [24]. These results show that an FSPM based on doped semiconductors can have the characteristics of fast modulation rate and simple control. At present, most of them belong to single-layer design, and the phase modulation is still smaller than 2π. The series connection of multilayer design can increase the phase modulation but still need to simplify the manufacturing process of multilayer compound semiconductors.

### 2.2 FSPM based on phase change materials

**Figure 3:**
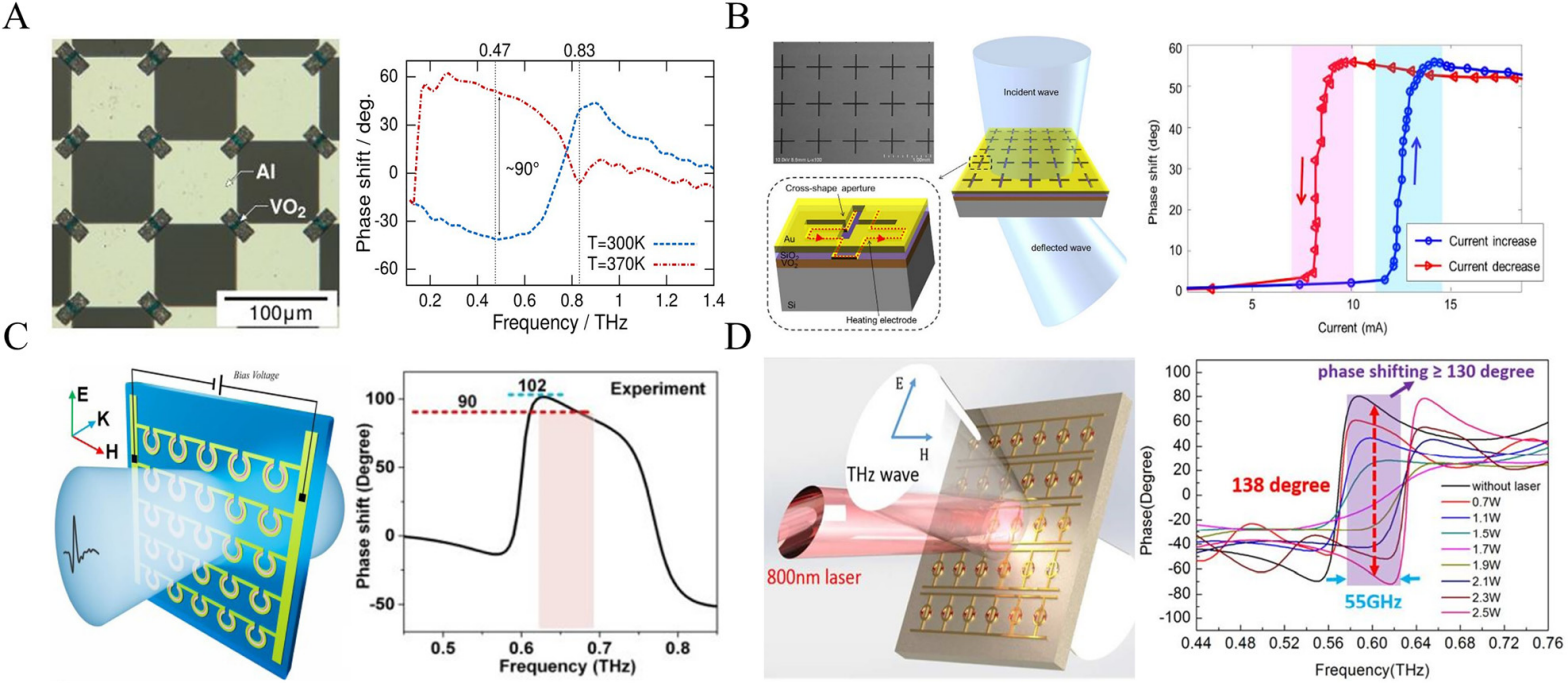
Structure diagram and phase modulation of FSPM based on phase change materials. (A) Micrograph of checkerboard nested VO_2_ terahertz phase modulator and its phase modulation results [25]; (B) structure diagram of electrothermal control terahertz phase modulator and its phase modulation results [26]; (C) structure diagram and phase modulation results of three concentric split ring phase modulators [28]; (D) diagram of ring dumbbell composite resonant phase modulator and phase modulation results with 138° [29].

Phase change materials that undergo dramatic changes in their properties when subjected to an external excitation are also popular research subjects for terahertz phase modulators. A key example is vanadium dioxide, which exhibits the characteristics of insulator-metal transition during the temperature-induced phase change process. This is conducive to changing the resonance characteristics of the composite metal structure, and has been widely used in terahertz modulation devices. Generally, the vanadium dioxide metamaterials mainly activate the phase change characteristics by changing the temperature of the material to achieve dynamic control. In 2016, Urade et al. developed a checkerboard-shaped nested VO_2_ terahertz phase modulator [25]. As shown in Figure 3A, the square metal aluminum films are alternately distributed in a checkerboard shape, and VO_2_ is nested in the diagonal corners of adjacent aluminum blocks. VO_2_ is in an insulator state at room temperature. When the temperature rises to 370 K, VO_2_ undergoes a solid–solid phase transition and becomes metallic. During the phase change of VO_2_, a modulation depth of 14 dB was obtained at 0.77 THz, and a 90° modulation phase was obtained at 0.42 THz. This temperature-controlled phase modulator has a good phase modulation range, but its temperature change is excited by an external laser. The entire modulation and excitation process is complicated, and is not convenient for real-time control. In response to this problem, Mohammed et al. proposed an electrothermally controlled terahertz modulator [26]. The VO_2_ film is combined with the complementary artificial microstructure. As shown in Figure 3B, a heating wire is placed between the metal structure and the VO_2_ film. The SiO_2_ film between the heating wire and the metal structure layer serves as an insulating dielectric layer. In the experiment, the external current fed into the heating wire gradually increased the temperature of the VO_2_ film in the area. During the transition of VO_2_ from a semiconductor state to a metallic state, a 57° phase modulation was observed. When the feed current gradually decreases, the temperature of the VO_2_ film also decreases, and it reversibly changes from the metal state back to the insulating state. The same modulation effect can be observed in this process. This electrothermal control method does not require an additional external laser, making the entire control process easier.

The phase modulation range is one of the most critical parameters of the phase modulator, and many achievements in improving the phase modulation range have been reported. Jae et al. proposed an active metasurface with nested VO_2_ microstructure in 2018. Here, the phase change characteristics of vanadium dioxide modulate the terahertz phase with resonance frequency tuning [27]. The metasurface resonance frequency moves from 0.52 THz to 0.37 THz with the varying input current. This shift in resonance frequency causes the phase modulation of the incident wave to be 64° at a frequency of 0.46–0.47 THz, with insertion loss of −6.5 dB. In 2021, Hu et al. proposed a terahertz amplitude and phase modulator based on VO_2_ reconfigurable metasurface. As shown in Figure 3C [28], the unit of the metasurface is composed of three concentric split rings on a sapphire substrate. By electrothermally triggering the insulator-to-metal transition (IMT) of VO_2_, the unit’s resonance mode, resonance intensity and surface current distribution be dynamically controlled. The experimental results show that under the applied current of 290 mA, the phase shift is 102° at 0.63 THz, and the modulation depth reaches 71% at 0.79 THz. A phase shift of more than 90° is modulated in a bandwidth of 70 GHz. In addition, in 2018, Zhang et al. proposed a ring-dumbbell composite resonator nested with VO_2_ nanostructures, as shown in Figure 3D [29]. This structure utilizes light control to achieve a large phase modulation. In this structure, a mixed-mode with enhanced resonance intensity is coupled by LC resonance and dipole resonance. The photo-induced phase transition characteristics of VO_2_ make it dynamically control the resonance intensity of this mode, resulting in a large phase shift of the incident terahertz wave, with a phase modulation of 138° at 0.6 THz. This is one of the results with the largest modulation phase in the current transmission metasurface. Phase change materials, especially VO_2_, whose electrical conductivity changes by several orders of magnitude, can significantly enhance the effect of phase modulation. However, its thermally induced characteristics lead to inevitable thermal residues, which limits the achievable modulation rate.

### 2.3 FSPM of liquid crystal

Liquid crystal is a substance that has both crystal and liquid properties in the molten state and retains the intermediate state between crystal and liquid. Liquid crystals have had a profound impact on optical display technology and the development of optoelectronic information technology. Different from compound semiconductors and phase change materials, the orientation of liquid crystal molecules changes under the force of an electric field, thereby changing the dielectric properties of liquid crystal materials. It is also a popular material for terahertz phase modulation [59], [60], [61].

**Figure 4:**
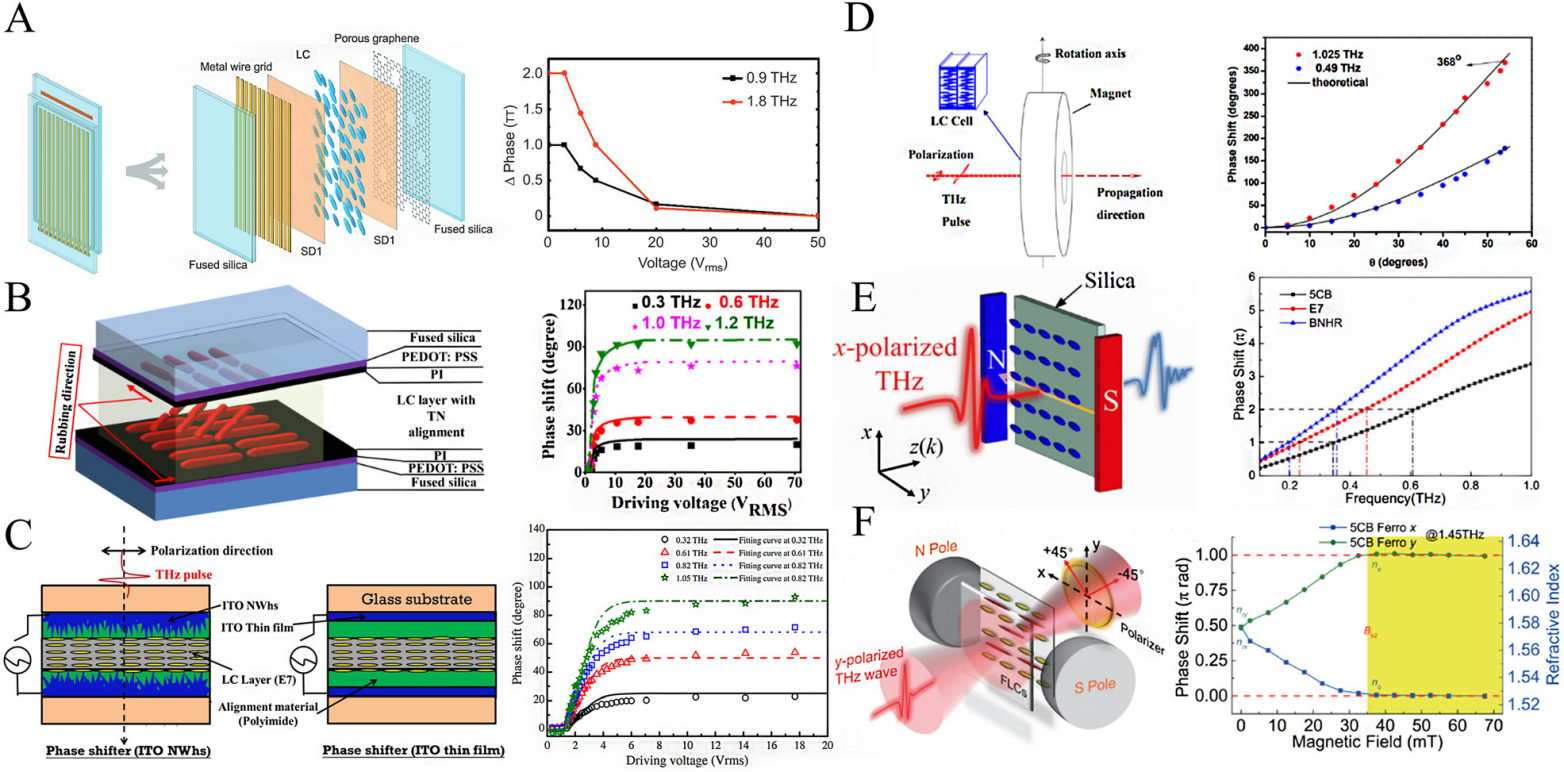
Schematic diagram of liquid crystal FSPM structure and phase modulation. (A) Schematic diagram of porous graphene electrode liquid crystal metasurface and phase change with voltage at 0.9 THz and 1.8 THz [63]; (B) schematic diagram of PEDOT/PSS electrode liquid crystal metasurface and phase modulation results [35]; (C) schematic diagram of indium tin oxide electrode liquid crystal metasurface and phase modulation results [40]; (D) schematic diagram of the magnetron terahertz phase modulator and its phase change with the angle of the external magnetic field at 0.49 THz and 1.025 THz [65]; (E) schematic diagram of phase modulation of 5CB, E7 and BNHR liquid crystals under an external magnetic field and the frequency response of these three liquid crystals under a 30 mT external magnetic field [66]; (F) the schematic diagram of the ferromagnetic liquid crystal terahertz phase modulator and its corresponding phase response under different external magnetic fields [67].

By processing the electrodes into different sub-wavelength structures, the formed liquid crystal metasurface can effectively tune the illuminated terahertz wave as an FSPM with low voltage and a large phase shift range [40, 62]. For example, Yang et al. combined the double-dipole metasurfaces with electrodes and successively reported reflective liquid crystal FSPM in the frequency range of 349–361 GHz [30], 325–337.6 GHz [31], 85–115 GHz [32]. The tunable phases are all above 300°. These phase modulators provide not only a wide range of phase modulation but also continuous adjustability in a wide frequency range. In related work, Ung et al. designed a new type of interdigitated electrode structure, which realizes the rapid switching between the in-plane and out-of-plane states of the terahertz wave. The device provides an actively controllable 35° phase delay at 2 THz, and it’s on/off cycle switching time is about 0.5 s, which is almost 100 times faster than the usual cycle time (over 40 s) [33]. Ji et al. proposed a dielectric metasurface with a linear complex lattice structure, which has polarization-dependent electromagnetic induction transparency (EIT) and artificial birefringence. Filling the liquid crystal on the dielectric metasurface offers a tunable phase shift of 60° under a bias electric field, which is a much larger phase range compared to filling the liquid crystal on a bare silicon wafer [34]. In addition, the electrode is combined with new materials such as graphene, indium tin oxide (ITO) nanomaterials to form a liquid crystal metasurface, increase the transmittance of the electrode and effectively improve the control performance of the electrode. Wang et al. utilized low-layer porous graphene as the electrode, as shown in Figure 4A, which greatly improved the modulator’s transmittance and achieved phase modulation greater than 180° above 0.9 THz and greater than 360° above 1.8 THz [63]. Li et al. utilized PEDOT:PSS thin layers to achieve a maximum phase shift of 360.5° at 1.68 THz and a maximum phase shift of 90° at 1.2 THz, respectively, as shown in Figure 4B. This device not only shows significantly lower threshold voltage and lower transmission loss under low driving voltage, but experiments also show the highest transmittance of 91.5 and 92%, respectively. Indium tin oxide nanowhiskers has excellent transmittance [64], and the required bias is as low as 2.6 V, which is compatible with CMOS technology. In 2014 and 2019, Yang et al. used ITO nanowhiskers as the interlayer of the transparent electrode and modulated the phase of 90° and 360° at 1 THz (as shown in Figure 4C), respectively [36, 40]. In addition to electrical control, the magnetic response of liquid crystals makes it possible to use an external magnetic field to control its optical properties. These devices have tunable characteristics without the necessary transparent electrodes and excessively high drive voltages. Chen et al. sandwiched E7 nematic liquid crystals to increase the interaction length and reduce the interface Fresnel loss, as shown in Figure 4D. With the addition of a continuously controllable magnetic field, the phase of the electromagnetic wave at 1 THz can be continuously tuned from 0° to 360° [65]. Yang et al. compared the phase response of three thermotropic liquid crystals (5CB, E7, and BNHR) to terahertz waves under an externally weak magnetic field (30 mT), and the results proved that the BNHR liquid crystal has a larger phase modulation depth and requires a lower magnetic field. As shown in Figure 4E, under the action of the initial magnetic field of 5 mT, the phase shift of the BNHR liquid crystal at 0.35 THz reaches 270° [66]. Ji et al. compared the birefringence characteristics of magnetic nanoparticles dispersed in liquid crystals at different concentrations and obtained a maximum phase shift of 180° at 1.45 THz (as shown in Figure 4F) [67]. The liquid crystal FSPM has the advantages of a large phase modulation range and flexible control but still has problems such as large insertion loss and slow response. Therefore, liquid crystal devices with low insertion loss and fast response need to be further explored.

### 2.4 FSPM based on two-dimensional materials

Two-dimensional materials, such as graphene [68], transition metal sulfide [69] and black scale [70], have unconventional optoelectronic advantages compared with bulk materials. Two-dimensional materials have rich physical behaviors, from broadband insulators to semiconductors, semi-metals, and even metallic properties, which can provide potential application values for various optoelectronic and photonic devices [71–73]. As the most representative of all two-dimensional materials, graphene is composed of a single layer of carbon atoms arranged closely in a hexagonal lattice, and it has a special zero bandgap energy band. Graphene has peculiar optoelectronic properties, such as good light transmittance, fast photoelectric response, ultra-high carrier concentration, ultra-wide spectral response, and strong optical nonlinear effects [74]. It is this series of advantages that make it unique in the development of high-speed terahertz phase modulators. The optical conductivity of graphene at terahertz frequencies is closely related to the Fermi energy. In practical applications, changing the Fermi energy of graphene causes the carrier concentration to change, which means that the conductivity can be controlled to adjust the phase characteristics of the terahertz wave [37, 75, 76].

**Figure 5:**
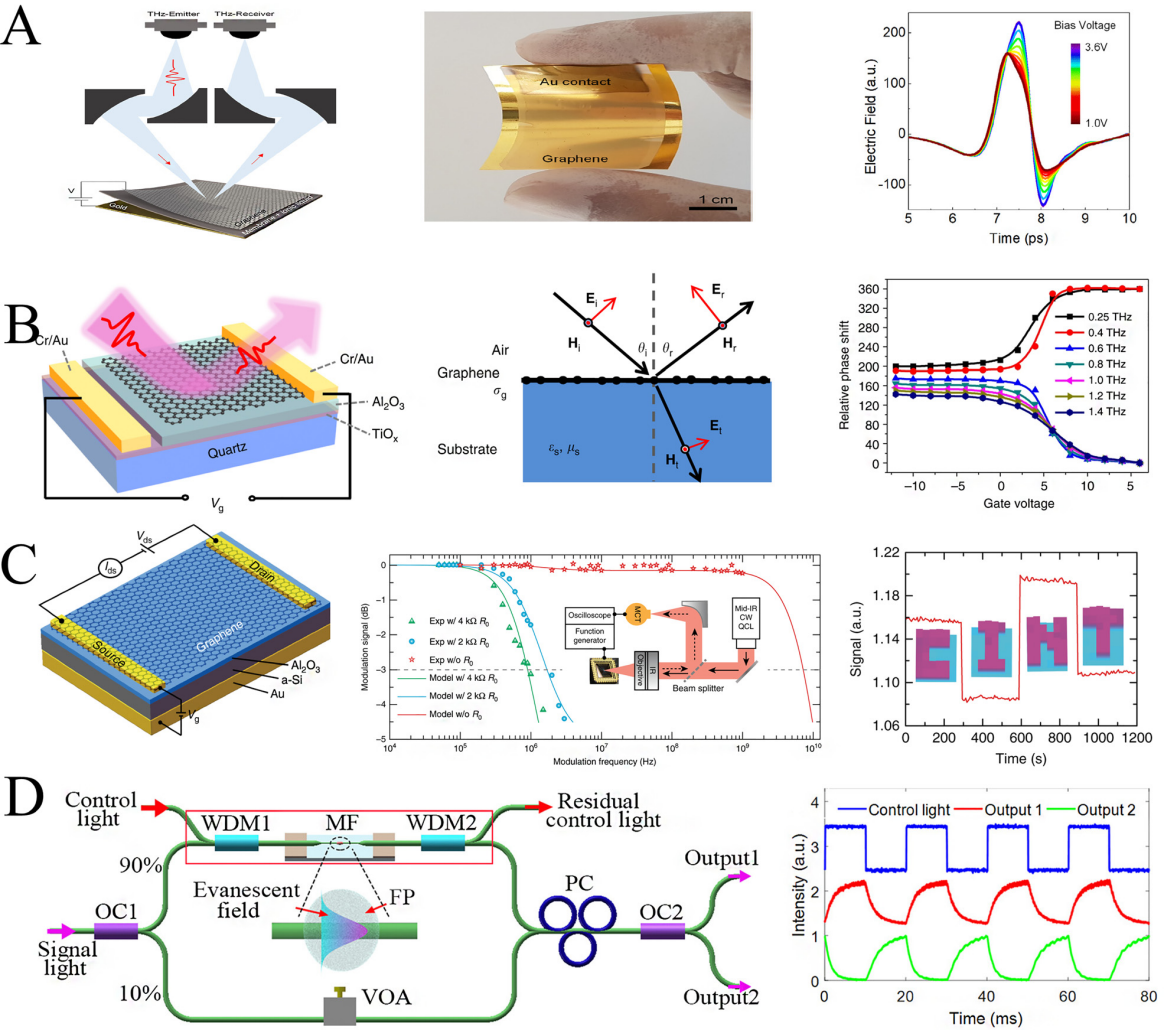
Structural diagram and phase modulation of two-dimensional material FSPM. (A) TDS experimental setup diagram, sample diagram and experimental results of graphene electronically controlled phase modulator [38]; (B) schematic diagram of broadband graphene phase modulator, schematic diagram of Brewster angle control, and experimental results of phase control at different frequencies [39]; (C) schematic diagram of low-voltage graphene metasurface, experimental diagram of modulation speed, and “CINT” spatial reflection mode [77]; (D) schematic diagram of experimental setup based on MZI all-light modulation, and measured waveforms of control light and modulated light [78].

As a terahertz phase linear tunable material, graphene was first proposed by Xiang Zhang in 2012. This integrated graphene metamaterial can modulate the phase over a range of 32.2° [37]. Since then, various graphene-based electronically controlled phase modulators have been reported. Kocabas et al. used graphene as a tunable impedance surface [38]. The graphene is placed at a quarter wavelength away from the reflective metal surface, and the surface impedance of the graphene is changed by applying a voltage to modulate the reflection phase. Figure 5A shows the experimental device and the experimental results obtained by the terahertz time-domain spectral reflectance measurement. The results show that a phase modulation of 180° is obtained at a single frequency point of 1.2 THz. However, the phase modulation of a single frequency point obviously cannot meet the needs of terahertz applications. Pickwell-MacPherson et al. utilized the phase jump property at the Brewster angle to achieve wideband phase modulation with a deep modulation range [39]. The reflected wave can be modulated by 180° when the p-polarized wave is incident at the Brewster angle. Based on this principle, the author rotates the Brewster angle from 65° to 71° by altering the surface conductivity of the graphene to modulate 140° phase in a wide frequency range of 0.5–1.6 THz, as shown in Figure 5B. The disadvantage is that its modulation speed is only 10 kHz. Chen et al. utilized an a-Si layer as part of the gate electrode of the hybrid graphene metasurface modulator to address the modulation speed of the graphene phase modulator, reducing the parasitic capacitance [77]. This work reduced the thickness of the insulating layer and successfully reduced the gate voltage to a few volts to regulate the conductivity of graphene. They formed a mixed graphene modulator into a pixel array and achieved 23 kHz single-pixel imaging through a series of spatial masks, as shown in Figure 5C. In addition to graphene, there are also related research reports on phase modulators based on other two-dimensional materials [78, 79]. In 2018, a Mach-Zehnder all-optical phase modulator based on two-dimensional fluorinated phosphine (FP) was achieved at an optical power of 290 mW [78]. The information carried in the control light is transmitted to the signal light through the mutual thermal optical coupling effect to varying the optical phase of the signal light. But like the vanadium dioxide FSPM, the modulation speed of this modulator is only 270 Hz due to the slow dissipation after the temperature rises, as shown in Figure 5D. Two-dimensional materials such as graphene provide new solutions for terahertz phase modulators, but the efficiency is still low. How to improve the interaction between graphene and terahertz and further improve the efficiency of this type of FSPM is the current problem to be overcome.

### 2.5 FSPM based on micro-electro-mechanical system

Micro-Electro-Mechanical System (MEMS) can reconfigure the unit cell geometry and is very attractive for terahertz phase modulation. MEMS technology has been developed for more than 50 years and has a wide range of applications in miniaturization, high-performance sensing, and micro/nano-level driving [80], [81], [82]. Different from semiconductors and phase change materials, phase modulators MEMS-based do not change material properties by external stimuli (such as electric field, light, heat or magnetic field). Instead, the MEMS is integrated into the composite unit structure of the THz metamaterial and by changing the mechanical properties and reconstructing the geometric structure to modulate the phase.

**Figure 6:**
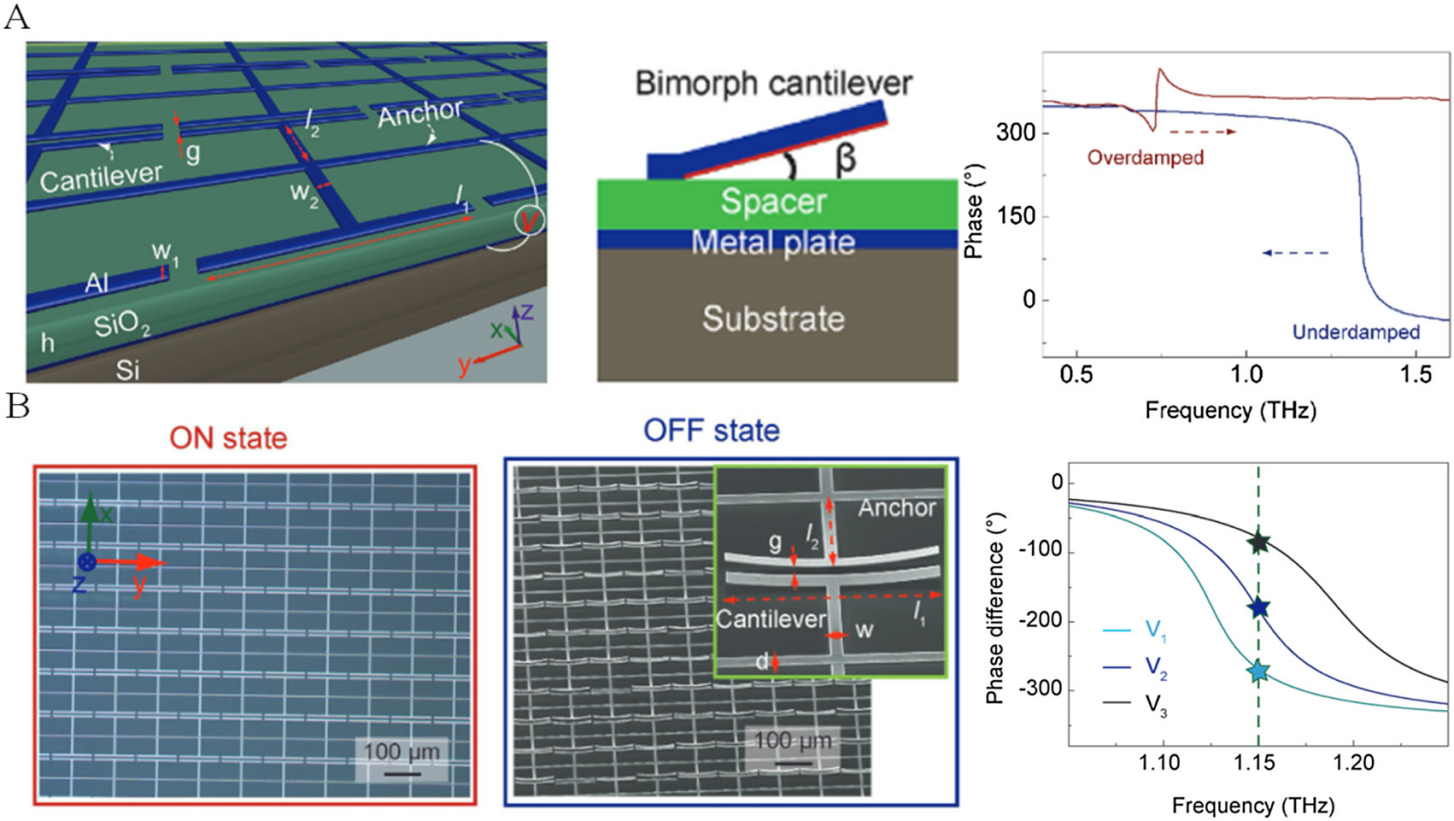
Schematic diagram and phase modulation of FSPM based on MEMS. (A) The schematic illustration of the MEMS-based phase modulator and the phase spectra operating in the “ON” and the “OFF” states; (B) the optical microscope and scanning electron microscope (SEM) images of the fabricated phase modulator array in the “ON” state and “OFF” state, and corresponding phase spectra.

In 2017, Cong et al. integrated an electrostatic cantilever into a metal-insulator-metal (MIM) metamaterial for dynamic phase modulation [83]. As shown in Figure 6A, the top of the phase modulator is composed of a cantilever array, the aluminum film on the silicon substrate serves as the bottom layer, and the middle layer is a stack of thick SiO_2_ layers, 50 nm thin Al_2_O_3_ layers, and a spacer. By applying a voltage between the bottom aluminum and the top cantilever resulting effective spacer thickness can be dynamically changed. They used MEMS technology to theoretically analyze the active phase transition in the MIM system from the perspective of phase span and resonance dispersion. The coupled-mode theory is used to analyze the reflection spectrum, where the inequality between the radiation and absorption quality factors determines the operating state. In addition, the dynamic phase transition driven by the cantilever in the modulator is explained from the mode expansion theory. In the same year, this research group further conducted research, the phase modulation span of the modulator working in the underdamped region covers the entire 360°, and the phase value at a fixed frequency can be tuned by changing the cantilever [84]. Continuous tuning of the cantilever structure by tuning the external voltage shifts the dipole resonance frequency excited by the y-polarization incident, resulting in continuous phase modulation, as shown in Figure 6B. The phase of the *x*-polarized wave remains fixed. In this way, the required phase difference is obtained at a specific operating frequency. The phase difference at 0.15 THz can be covered −75° to −340°. By increasing the initial height of the suspension cantilever, a larger frequency shift can be provided for the underdamped region, and the phase modulation range can be further expanded. In addition, the modulation of the orthogonal polarization phase difference realizes the active modulation of the output polarization state from the right circularly polarized (RCP) to the linearly polarized (LP) and then to the left circularly polarized (LCP). This MEMS-based FSPM can actively obtain three typical output polarization states. The general elliptical polarization can also be obtained by continuously modulating the external voltage. This MEMS-based device provides the possibility for local control in each unit of metasurface and provides an unprecedented technological approach for independent control to achieve more complex functions. With the development of more stable and more precise micro-cantilevers or nano-cantilevers, the combination of MEMS technology with metamaterials will effectively promote the advancement of higher-precision terahertz phase modulators.

## 3 Terahertz guide wave phase modulator

Terahertz FSPM combined with different materials indicate favorable phase modulation, but free-space terahertz wave needs to be vertically or obliquely incident to stimulate the corresponding response. This quasi-optical operating model brings huge obstacles to system integration, especially for small and portable systems. Therefore, for the application of an integrated terahertz system, the design of an integrated phase modulator is indispensable. Terahertz integrated phase modulators are mainly divided into two categories: monolithic integrated and waveguide embedded. The former mainly includes high-low pass type, reflection type, and vector synthesis type. For the high-low pass and reflection approaches, it is easy to realize significant phase modulation, but they have low fault tolerance and high requirements for capacitance and inductance. Especially in the terahertz band, their parasitic capacitance and inductance have a significant impact on the device’s performance. The vector synthesis phase modulator realizes phase modulation by controlling the amplitude of two orthogonal signals output by the coupler. The phase modulation angle is large, but the circuit structure is very complex. The latter is mainly to embed tunable electronic components (MEMS, reactance components) in the waveguide, changing the dispersion characteristics of the waveguide to modulate the phase. This type of phase modulator is simpler than a monolithic integrated modulator. It has the characteristics of modular integration, but it has higher requirements for cavity processing accuracy, and the actual processing yield is a current problem. In addition, since most of the waveguide-embedded phase modulators mainly use MEMS for mechanical modulation, there is also a sacrifice in the phase modulation rate. The following is a review and summary of these two approaches for terahertz guide wave modulators.

### 3.1 Monolithic integrated phase modulator

At present, the terahertz monolithic integrated phase modulator mainly adopts a vector synthesis scheme. The basic principle is to superimpose orthogonal signals of different amplitudes to modulate the terahertz phase by utilizing the amplification characteristics of active devices. It is generally composed of a quadrature generating network, a variable gain amplifier (VGA), and a vector synthesis network. The input signal is first split into orthogonal I channel and Q channel through the quadrature generating network. Then, the VGA selects the two orthogonal signals in amplitude and polarity. Finally, the two signals are summed by the vector synthesis network to create a synthesized output signal with variable phase [85].

**Figure 7:**
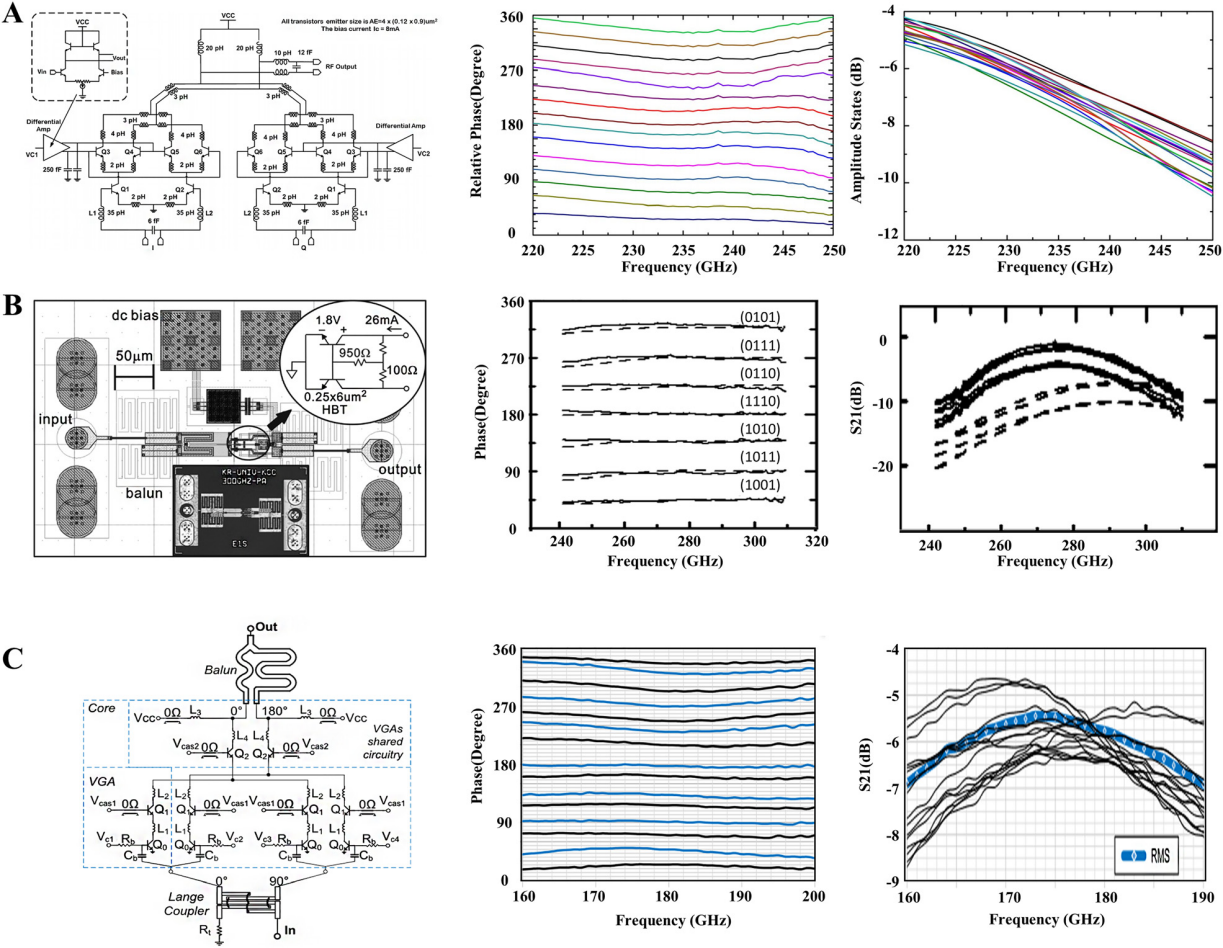
Schematic diagram and phase modulation of monolithic integrated phase modulator. (A) Schematic diagram of single Gilbert unit terahertz phase modulator based on 250 nm InP DHBT technology, phase modulation and loss results [41]; (B) schematic diagram of three-stage buffered terahertz phase modulator and its phase and insertion loss [42]; (C) schematic diagram of low power vector terahertz phase modulator and its phase and insertion loss [47].

Generally, each VGA is implemented using a Gilbert unit with four sub-units. In this way, eight units can be combined at the differential output node to match the impedance relationship. However, reducing the node impedance also increases the complexity of the device and unnecessary parasitics. In order to solve this problem, in 2015, Kim et al. developed a single Gilbert unit terahertz phase modulator based on 250 nm InP DHBT technology [41], as shown in Figure 7A, which consists of a quadrature generating network, VGA, and output Balun composition. Different from traditional VGA that includes dual Gilbert units, this VGA uses a single Gilbert unit to achieve broadband impedance matching. The Balun combines the two output signals from the vector modulator while suppressing their common-mode components to obtain an appropriate phase. In addition, a loss matching resistor is inserted at the output of each unit to improve the working bandwidth and circuit stability. In the entire WR-3 frequency band, 220–320 GHz, a 360° continuous phase modulation is realized, with an average insertion loss of 13.7 dB and a maximum phase error of 10.2°. This terahertz phase modulator implemented by a single Gilbert unit has been partially simplified in structure, which reduces the overall parasitics and circuit complexity. However, there is still some impedance mismatch between the output Balun and the cascaded amplifier, which produces ripples in the frequency response of the phase modulator resulting in a large offset phase error. To solve the phase error caused by the matching ripple, Quan et al. connected the input and output buffer amplifiers to a single-stage VGA and formed a three-stage design [42]. This design makes the input and output impedance unaffected by DC bias to guarantee a certain operating bandwidth and improve impedance matching by increasing the switching ratio and reducing matching ripple. As a result, the phase shift accuracy improved to 3.6°. In this device, the insertion loss is about 10 dB, as shown in Figure 7B. In order to further reduce this loss, Testa et al. have proposed a low-power vector phase modulator [47]. The chip model and measured results are shown in Figure 7C, which is on the circuit composed of Lange coupler, variable gain amplifier (two pairs of VGAs), and Balun. The circuit integration area is only 0.07 mm^2^ and the average DC power consumption is only 12.4 mW. The single-ended input outputs two I/Q signals with a phase difference of 90° through the Lange coupler. The two pairs of VGAs control the amplitude of the front-stage input signal in quadrature. An additional 180° phase mix achieves 360° phase coverage. In the VGAs circuit design, a single-ended Gilbert unit is used to avoid the unnecessary loss caused by Q_0_ being distributed to two transistors. The peak gain technology increases the gain of the VGAs by 3 dB and modulates the 360° continuous phase at 160–190 GHz. The insertion loss under the test 16 phase shift states is better than 9 dB, the phase root mean square error is less than 8°, and the minimum root mean square insertion loss at 174 GHz is 5.5 dB. The MIPM based on vector synthesis has a good phase modulation range, but its structure is complicated, the insertion loss is large, and the cost is still high. These are the biggest problems encountered at present.

### 3.2 Waveguide embedded phase modulator

**Figure 8:**
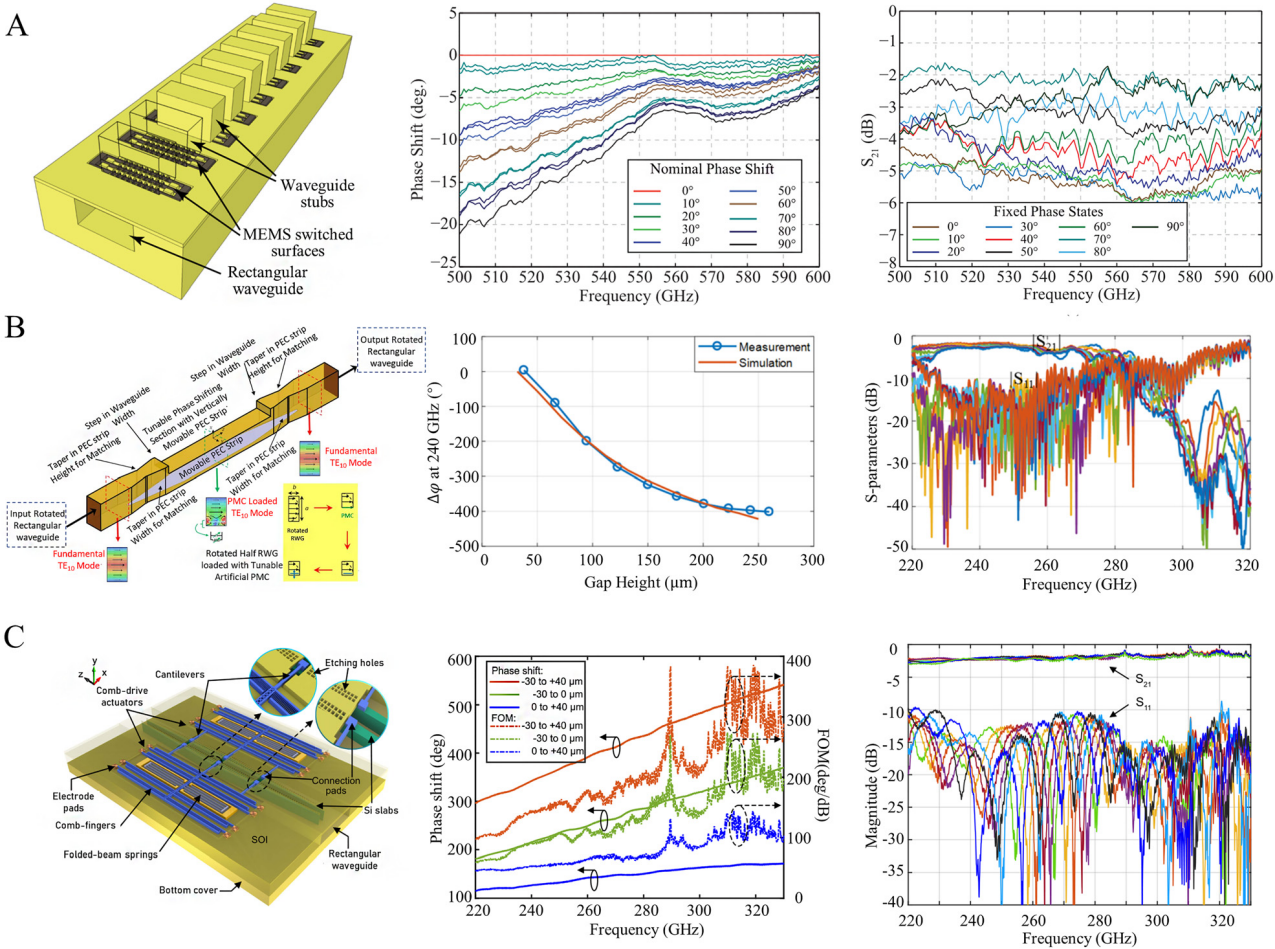
Structure diagram and phase modulation of waveguide embedded phase modulator. (A) Schematic diagram of rectangular waveguide embedded phase modulator based on MEMS and its phase and insertion loss [86]; (B) schematic diagram of MEMS parallel metal plate rectangular waveguide phase modulator and its phase and insertion loss [45]; (C) schematic diagram of waveguide embedded terahertz phase modulator with super-couple mode and its phase and insertion loss [48].

For the rectangular waveguide embedded phase modulator, Shah et al. proposed a MEMS-based reconfigurable modulator in 2015 [86]. This modulator consists of a series of MEMS, a rectangular waveguide, and a series of E-Plane waveguide piles containing a set of fixed vertical cantilevers and a set of movable vertical cantilevers as shown in Figure 8A. When the MEMS reconfigurable surface is open, there is a gap between the vertical cantilevers, and electromagnetic waves propagate into the waveguide pile. The MEMS mechanical actuator controls the moving vertical cantilever to move in the horizontal direction. When the two vertical arms touch, a closed surface is formed, preventing electromagnetic waves from propagating into the E-plane waveguide pile. The results show that the linear phase shift is 20° in ten discrete states at 500–550 GHz, the corresponding insertion loss is better than 3 dB, and the return loss is better than 15 dB. In 2018, Anokiwave et al. developed a phase modulator in a rectangular waveguide, also with MEMS components [45]. As shown in Figure 8B, the structure is inserted into the narrow side of the rectangular waveguide with parallel metal plates. The parallel metal plate is equivalent to a perfect magnetic boundary loaded into the waveguide. The width of the metal plate and the distance from the narrow side affect the wave impedance and propagation constant. The MEMS voltage-controlled drive is used to vertically move the distance between the metal plate and the narrow side to vary the wave impedance and propagation constant. The change of dispersion relation of rectangular waveguide causes the phase modulation of electromagnetic wave transmission. Finally, in the range of 230–250 GHz, the modulation phase reaches 380°, the insertion loss is better than 3 dB, the average insertion loss is 2.25 dB, and the return loss is better than 10 dB. In 2019, Aidin et al. reported a new finger phase modulator based on SOG (silicon-glass) technology [87]. The high-resistivity silicon (HRS) waveguide is loaded with finger-shaped high-conductivity silicon (HCS), and the micro-positioner moves the finger-shaped high-conductivity silicon (HCS) above the waveguide to perturb the waveguide field to modulate the terahertz phase. In addition, a waveguide embedded terahertz phase modulator with supermode propagation has been reported [48]. As shown in Figure 8C, the phase modulator combined with Silicon-On-Insulator (SOI) micromachining technology integrates two symmetrical parallel tapered silicon wafers in a rectangular waveguide. By control the MEMS comb driver to change the coupling distance between the silicon chips, the effective refractive index of the supermode is tuned, thus inducing phase modulation. When the silicon board is offset by −30 µm and 40 µm, the maximum continuously adjustable phase is 550° in the entire passband of 320–330 GHz. The worst insertion loss is 2.5 dB, the average insertion loss is 1.94 dB, and the phase error is 4°.

In addition to MEMS, graphene and liquid crystal materials are also applied in waveguide embedded phase modulators. Mittendorff et al. reported a tunable absorber in which a rectangular waveguide is embedded in a graphene sheet. The carrier concentration in the graphene sheet is changed by applying a gate voltage to achieve a modulation effect and accompanied by a phase modulation [88]. The authors designed two structures with graphene sheets loaded on the bottom of the waveguide and the center of the waveguide. The experimental results show that the modulation depth is about 90% from 0.15 THz to 0.7 THz. The structure with graphene embedded on the bottom of the waveguide can achieve a modulation depth of 50% at 0.2 THz, and the maximum phase modulation is 30° at 0.7 THz. Polat et al. reported a continuously adjustable non-radiative dielectric waveguide (NRD) phase modulator based on liquid crystal technology at 98–110 GHz [89]. Compared with other dielectric waveguides prone to radiation loss, non-radiation dielectric waveguides will not radiate out even at bends and discontinuities. This structure embeds liquid crystal in the NRD, controls the dielectric constant of the liquid crystal by the external voltage, and changes the dielectric constant to obtain a phase modulation. It can get a maximum phase modulation of 280° under a bias voltage of ±150 V, an insertion loss between 2.9 and 4.9 dB, and a maximum quality factor of 85°/dB. The waveguide-embedded phase modulator has good phase efficiency, but its rate of electromechanical control is relatively slow. Therefore, how to increase the modulation rate is a direction for further research and exploration of this type of phase modulator.

### 3.3 Meta-chip phase modulator

**Figure 9:**
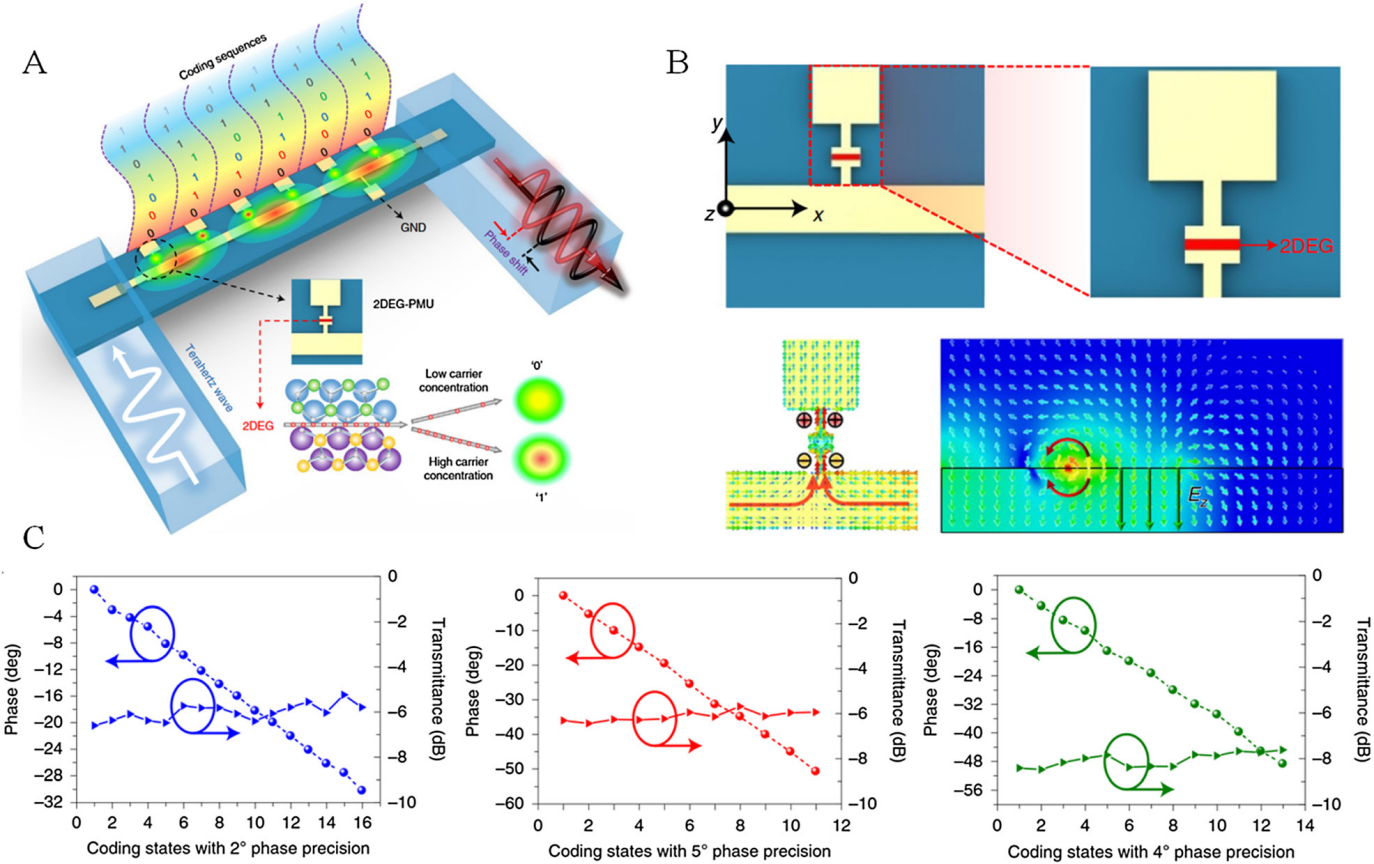
Structure diagram and phase modulation of Meta-chip phase modulator. (A) Schematic diagram of multi-channel field perturbation terahertz phase modulation meta-chip; (B) phase modulation with an accuracy of 5° and 6.14 dB insertion loss under different codes [90].

The terahertz FSPM based on metasurface has a simple structure with low phase modulation accuracy and large insertion loss. The terahertz GWPM has high precision, but its structure is complex. How to combine the advantages of the two is an important research direction. Recently, Zhang et al. proposed a multi-channel field perturbation terahertz phase modulation meta-chip [90]. The modulator combines the resonant characteristics of the microstructure unit in the FSPM with the monolithic integration process. It has a very simple topology and high integration. High precision phase modulation is realized by coding the unit in the meta-chip (as shown in Figure 9A). The meta-chip comprises a two-dimensional electron gas perturbation microstructure unit (2DEG-PMUs) and microstrip transmission line (as shown in Figure 9B). Each 2DEG-PMU consists of a metal block, an open thin metal wire and two metal electrodes located on both sides. The 2DEG adopts the same manufacturing process as the traditional high electron mobility transistor technology. It is embedded in the opening of the microstructure to provide tunable characteristics. The terahertz wave is weakly resonant coupled with the microstructure in the chip, and the external bias voltage controls the 2DEG concentration changing the coupling strength, which modulates the phase of the propagating terahertz wave. The combination and arrangement of multiple 2DEG-PMUs can form rich perturbation states. Each 2DEG-PMU on the meta-chip shows the perturbation resonance states of “0” and “1”, respectively by different bias voltages. These perturbations are superimposed and coupled, resulting in the nonlinearity of the overall phase shift. This nonlinearity enriches the phase shift coding database and dramatically improves the accuracy of phase shift. Their experimental results show that (as shown in Figure 9C), it has a high phase modulation accuracy of 5° at 0.265 THz. The average error is only 0.35°, and the average insertion loss is as low as 6.14 dB. Moreover, the amplitude fluctuation is only 0.5 dB. Compared with the traditional metasurface, this work ensures high-precision phase modulation with only slight amplitude fluctuation. Compared with monolithic integrated phase modulator and waveguide embedded phase modulator, the whole topology is very simple without complex structure design, which significantly reduces the parasitic effect in the terahertz band. However, the range of phase modulation is small. Realizing large-scale phase modulation and expanding to a higher frequency band are the key points to be overcome in future research.

## 4 Functional devices derived from terahertz phase modulation

**Figure 10:**
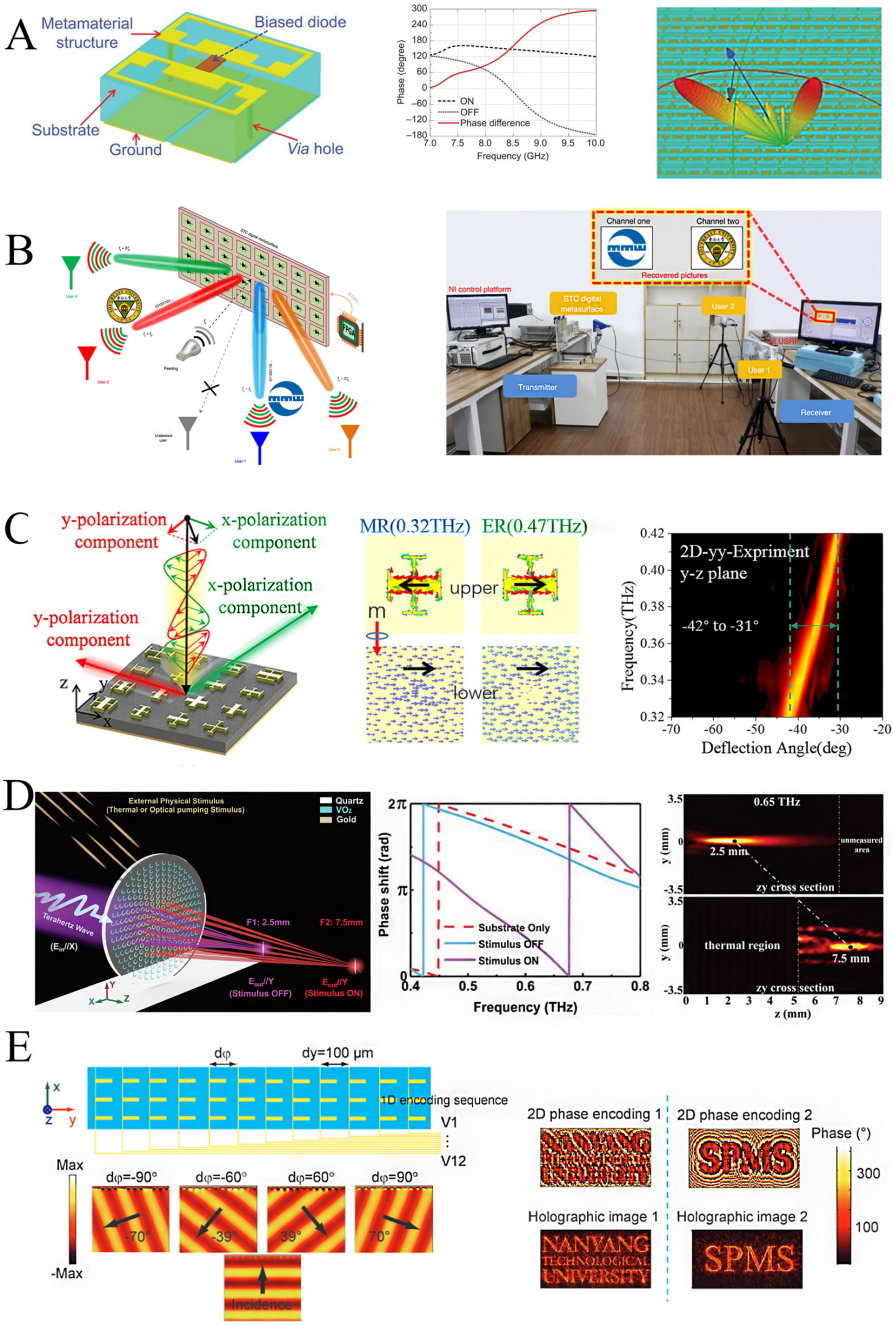
Various functional devices are derived from terahertz phase modulation. (A) Coding metasurface unit structure and its phase modulation results and beam splitting manipulation [57]; (B) space division multiplexing and frequency division multiplexing wireless communication system based on space–time coding metasurface [95]; (C) schematic diagram of terahertz polarization beam splitting based on anisotropic metasurface, current distribution of phase modulation unit and experimental results of polarization beam splitting [56]; (D) schematic diagram of planar metalens based on a phase gradient vanadium dioxide composite metasurface, the result of phase modulation and lens focal length switching [98]. (E) Programmable metasurface based on MEMS for beam steering and hologram [84].

As one of the essential attributes of electromagnetic waves like amplitude, polarization, and frequency, phase modulation plays a critical role in communications, detection, and imaging applications. The phase gradient and coding metasurface realize the functions of traditional high-complexity devices with a more flexible, more compact, and less complex structure. Functionalities include beam scanning, terahertz polarized beam splitter, zoom lens, and intelligent metasurface [91, 92]. In 2014, Cui proposed a programmable metasurface, which encodes a phase modulator with a reflection phase response of 0° and 180° according to a certain sequence, as shown in Figure 10A. Quantifying the metasurface phase simplified the design process and complexity, and its flexible wavefront control capability is verified in the microwave range [57]. The designed unit is a reflective phase modulator in which the PIN diode is mounted between two plane symmetrical metal patches on the top layer. The external voltage controls the state of the PIN diode to switch between “ON” and “OFF”. The two different states of the diode code as “0” and “1” respectively. The device allows flexible conversion from scattered beams to dual beams by different sequences of codes. Subsequently, the convolution theorem was combined with the beam deflection theory to realize the function of steering the scattered beam to any direction [71]. In addition to applications in the space domain, related studies have been reported in the time domain and frequency domain. In 2015, Hadad et al. proposed the concept of space–time gradient metasurface [93]. By controlling different spatial positions on the metasurface to switch periodically or non-periodically “on” and “off” in the time domain, this device provides rich degrees of freedom for the flexible manipulation of the beam. On this basis, Zhang et al. designed a space–time coding metasurface using time modulation of reflection coefficients which manipulate the spatial and spectral characteristics of electromagnetic waves by cyclically switching the coding sequence [94]. The same team optimized the coding matrix based on the space–time coding metasurface and directly loaded the information on electromagnetic wave spatial and frequency spectrum characteristics, forming a new wireless communication method of space division multiplexing and frequency division multiplexing [95]. According to the number of target users and the spatial location, the direct information coding scheme designed in advance can simultaneously and independently carry out real-time information transmission with multiple users. This method does not require a digital-to-analogue conversion and mixing process (as shown in Figure 10B). And at the same time, it has the desirable secure communication characteristics of directional modulation. Each designated user has its independent receiving channel through a specific harmonic frequency. Other unwanted users of the direction cannot recover the correct information due to the characteristics of the directional modulation. In the past research, many metasurface holograms were usually used to record the amplitude and phase information of light to display images [96, 97].

Compared with traditional computer-generated holograms, metasurface holograms have the advantages of low noise, high efficiency, and good image quality. Li et al. realized multiple required holographic images based on a 1 bit programmable metasurface. Different from the traditional static metasurface holography, this metasurface containing the phase modulation unit realizes the dynamic reconstruction of holographic imaging. By applying an electrically controlled diode to the metasurface, the phase of each unit can be modulated by different bias voltages. The theoretical reflection efficiency is about 90%, and the reflection phase modulation exceeds 180° [52]. Based on this idea, in the terahertz frequency band, terahertz programmable metasurface based on complementary metal-oxide-semiconductor (CMOS) chip technology was fabricated [99]. The array consists of 576 phase modulation units. Each unit is individually addressable and digitally programmable, with 8 bit GHz speed control. According to the corresponding coding sequences, this kind of terahertz programmable metasurface can realize amplitude modulation, ±30° dynamic beam scanning, multi-beam forming, and programmable holographic projection. The combination of phase modulation and polarization control can also split the terahertz polarization beam. In 2019, Zeng et al. integrated the phase information of different polarization components into a matrix, separating the different polarization components into orthogonal planes with different angles [56]. Different from the traditional one-dimensional metasurface, as shown in Figure 10C, this subarray contained different polarization phase information. By varying the phase information of different polarizations in the matrix, the phenomena of abnormal reflection and separation of terahertz dual-polarization based on the anisotropic matrix metasurface are proved theoretically and experimentally. In 2020, Kou et al. proposed planar metalens based on a phase gradient metasurface composite with vanadium dioxide [98]. This metalens is controlled by external thermal source to switch the focal length (as shown in Figure 10D). By utilizing the insulator–metal phase transition characteristics of the vanadium dioxide component, the composite metasurface exhibits different phase gradient distributions, resulting in different focal points. Both simulation and experimental results show that vanadium dioxide behaves as a dielectric to terahertz waves in the absence of external excitation. When an external thermal stimulus is applied, vanadium dioxide changes from an insulating to a metallic state. In the two cases, the phase modulation units on the metasurface respectively show different phase distribution responses, achieving variable focus. In 2017, Singh et al. demonstrated the programmable metasurface based on MEMS technology for terahertz beam deflect and hologram [84]. As shown in Figure 10E, by performing corresponding 1-dimensional encoding on the angle of the cantilever, the metasurface responds to different phase gradients to deflect the terahertz beam with different angles. In addition, a 2D coding sequence was designed for the terahertz hologram, and the dynamic hologram was successfully realized. In addition, there are many research developments on the application of terahertz phase modulation in electromagnetic beam control, which are described in detail in the review [100]. There are also reconfigurable metasurface beam manipulate technologies, such as thermally or electrically tunable materials (such as VO_2_, LC, and graphene) and MEMS technologies, and some new approaches have been widely used in applications such as beam polarization and beam scanning [11, 26, 84, 101]. The review pointed out that integrated circuit technology, as a classic technology, can develop phase shifters with stability and high switching speed. The reconfigurable metasurface technology is also very attractive, especially the field-programmable metasurface with real-time beamforming [57]. However, due to the complexity of the feeding circuit, it is currently difficult for this type of metasurface to individually modulate the phase response in the terahertz frequency band. The on-chip design scheme utilizing CMOS or MEMS technology to integrate metasurfaces is expected to be applied to realize the integrated manufacturing of terahertz beam steering devices [80, 102].

## 5 Conclusions and prospects

**Figure 11:**
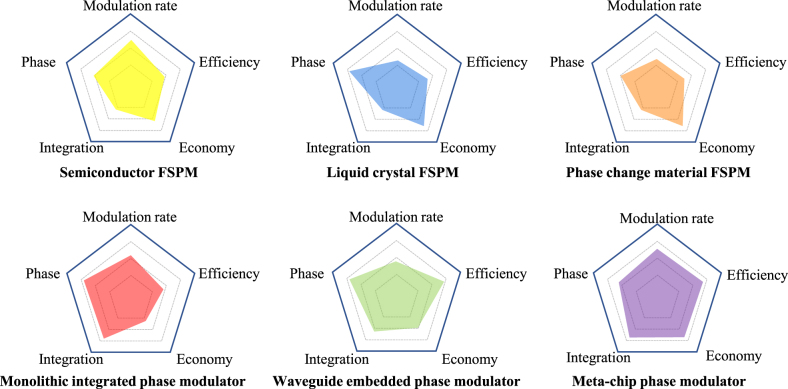
Comparison of characteristics of different types of terahertz phase modulators.

In summary, we briefly reviewed the development of terahertz phase modulators from two aspects of FSPM and GWPM. It involves the cross-fusion of multiple materials and processes. FSPM exploits materials such as doped semiconductors, phase change materials, liquid crystals, and graphene. The change of the resonance characteristic causes the phase modulation of FSPM. That is, the amplitude and the phase are related to each other. According to the KK relationship, the most significant phase jump occurs at the deepest amplitude. The greater the phase, the greater the loss. Therefore, the modulation efficiency of the FSPM is not high generally (as shown in Figure 11). It is a feasible solution to adopt the cascade method. Each stage modulates a particular phase with a small amplitude change, and the multi-stage interconnection increases the modulation phase. Of course, this method also has the problem of increased insertion loss caused by the increase in the number of layers. Therefore, selecting a substrate with a minor loss to design a multilayer structure is a key challenge for the terahertz FSPM. In addition, different materials exhibit differentiated phase modulation effects. For example, doped semiconductors have higher carrier concentration and electron mobility providing higher modulation rates. The long recombination time of liquid crystals and the problem of thermal retention of phase change materials all lead to a relatively slow modulation rate. However, the liquid crystal material varies the relative permittivity to directly affect the phase velocity of the terahertz wave propagation, providing a greater phase modulation.

Generally, the current terahertz phase modulator still has the problems of small phase modulation range, large loss and high power consumption. Fortunately, a lot of excellent work is overcoming the above-mentioned problems. For example, the Pancharatnam–Berry phase of spatial variation can be used to obtain an additional 180° phase modulation, which can improve the coverage of phase modulation while guaranteeing a certain loss [103–105]; apply high-process chip integration technology to reduce the packaging area of the phase modulator and reduce the power consumption [47]; another issue of great concern is the stability of the modulator. Currently, most terahertz phase modulators are still in the laboratory stage. One of the very important reasons for this is its weak anti-interference capability and poor stability. Conventional methods to prepare terahertz phase modulators are very sensitive to material defects and physical bending. Recently, a terahertz chip with topological photonics has been effectively solved this problem, which has three major properties such as robustness, single-mode transmission, and linear dispersion, and exhibits excellent transmission even after propagation through sharp corners [106]. Applying such topological photons to develop terahertz phase modulator designs will be expected to improve the stability of terahertz phase modulators and facilitate the commercial application of terahertz phase modulators as well as other terahertz devices.

Compared with the FSPM, the GWPM generally shows better performance. The monolithic integrated phase modulator draws on the design method of microwave and millimeter waves and has good modulation rate, phase modulation, and integration. However, the very prominent point is that the Si-based COMS and InP HBT technologies used in the terahertz phase modulators still have significant losses. In addition, the complex circuit structure also introduces inevitable parasitic effects in the terahertz frequency band. These are also problems that need to be overcome in the follow-up development of monolithically integrated terahertz devices. The waveguide embedded phase modulator has high integration, and its phase modulation and control efficiency are ideal. However, the current technologies are all based on mechanical control, so the modulation rate remains its biggest shortcoming. Therefore, how to develop high-speed terahertz mechanical switches is a complex problem for waveguide embedded modulators. In addition, the structure of the waveguide embedded modulator is also relatively complicated, which poses many challenges to the processing technology of the cavity and the assembly of the device. Compared with monolithic integration and waveguide embedded phase modulators, the novel meta-chip phase modulator has a very simple circuit topology and structural design while offering the advantages of high speed, high efficiency, and high integration. This design method has opened up a new idea for developing terahertz phase modulators. However, the phase modulation range of the current meta-chip phase modulators needs to be further improved, which is also the focus of the subsequent development of such devices.

**Figure 12:**
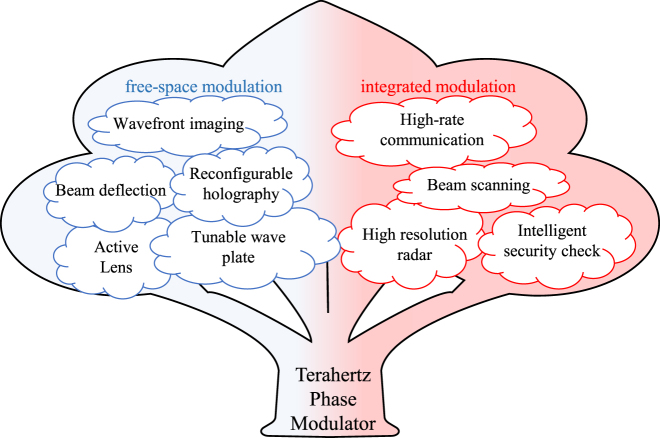
Application and prospect of terahertz phase modulator.

As mentioned above, compared with microwave and millimeter waves, terahertz has higher beam directivity, faster communication speed, better resilience against interference, and higher imaging resolution. It highlights its substantial application potential in the fields of communication, radar, and imaging. Different types of terahertz phase modulators can be used in different systems, as shown in Figure 12. For example, the transmission FSPM can be used in wavefront imaging systems and tunable lenses and wave plates in the terahertz optical path. The reflective FSPM is combined with a digital control circuit to form a terahertz smart metasurface, which is used to improve the communication quality. The GWPM can be used as a high-order modulator to increase the transmission rate of terahertz communication. It can also constitute a terahertz phased array by a multi-channel combination for communication tracking and aiming high-resolution radar. In addition, the phase modulator will derive more and more abundant functions and features in the future. For example, utilizing terahertz phase modulators to engage in nondestructive testing or biodetection, terahertz FSPM can be flexibly assigned with different wavefront phase information, which can provide more analytical dimensions for terahertz-matter interaction to improve detection accuracy. The high-speed terahertz phase modulator array may replace the traditional mechanical chopper to achieve high-speed phase matrix transformation, which combined with additional matrix algorithms improves the imaging resolution and signal-to-noise ratio for the imaging system. Phase modulator arrays can also provide richer dynamic modes, allowing multiple measurements or detection modes to the same object, potential means to improve the accuracy and efficiency of detection or imaging. In addition, terahertz phase modulators can be combined with advanced artificial intelligence and deep learning algorithms to achieve adaptive phase corrections and phase compensation, providing important technical support for terahertz communications, nondestructive testing, imaging systems, and biological detection, and making terahertz systems toward adaptive and intelligent evolution.
